# Supplementary material for: The interactome of intact mitochondria by cross-linking mass spectrometry provides evidence for coexisting respiratory supercomplexes
Source: Mol Cell Proteomics. 2017 Dec 8;17(2):216–32. doi: 10.1074/mcp.RA117.000470 (PMC5795388; doi:10.1074/mcp.RA117.000470)
Supplement: Supplemental Data [file supp_RA117.000470_133922_0_supp_23978_fzffwf.zip › spectra_annotation/mito_spectra_annotation/67-1-14-1-17-1.pdf]

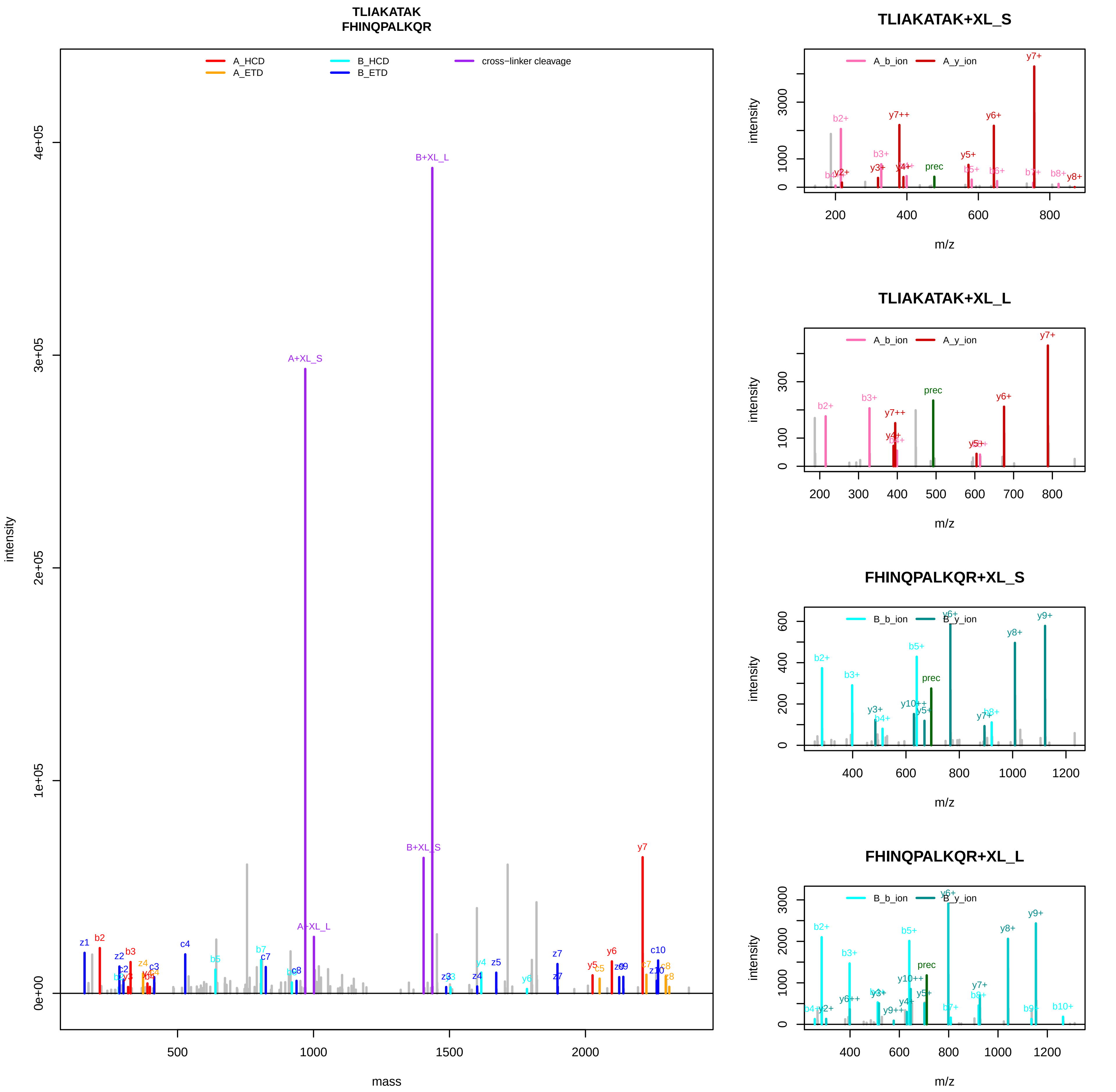
TLIAKATAK
FHINQPALKQR
A_HCD
A_ETD
B_HCD
B_ETD
cross−linker cleavage
B+XL_L
A+XL_S
B+XL_S
A+XL_L
y7
y6
y5
c10
c7
c8
z7
z5
z4
y4
z3
c8
b7
c7
b5
c4
z1
b2
z2
b3
c2
c3
z4
intensity
mass
0e+00
1e+05
2e+05
3e+05
4e+05
500
1000
1500
2000
TLIAKATAK+XL_S
A_b_ion
A_y_ion
y7+
b2+
y7++
y6+
b3+
y5+
y3+
y2+
prec
b8+
y8+
intensity
m/z
0
1000
3000
200
400
600
800
TLIAKATAK+XL_L
A_b_ion
A_y_ion
y7+
b2+
b3+
y7++
prec
y6+
intensity
m/z
0
100
300
200
300
400
500
600
700
800
FHINQPALKQR+XL_S
B_b_ion
B_y_ion
y6+
y9+
y8+
b2+
b3+
b5+
prec
y3+
b4+
y10++
y5+
y7+
b8+
intensity
m/z
0
200
400
600
400
600
800
1000
1200
FHINQPALKQR+XL_L
B_b_ion
B_y_ion
y6+
y9+
b2+
y8+
b5+
b3+
prec
y10++
y6++
y3+
y4+
y5+
y7+
y2+
y9++
b7+
b9+
b10+
intensity
m/z
0
1000
2000
3000
400
600
800
1000
1200
